# Restriction of Manganese Intake Prevents the Onset of Brain Manganese Overload in *Zip14*^−/−^ Mice

**DOI:** 10.3390/ijms22136773

**Published:** 2021-06-24

**Authors:** Yuze Wu, Guojun Wei, Ningning Zhao

**Affiliations:** Department of Nutritional Sciences, The University of Arizona, Tucson, AZ 85721, USA; yuzewu@email.arizona.edu (Y.W.); gwei@email.arizona.edu (G.W.)

**Keywords:** ZIP14, *SLC39A14*, manganese, metal metabolism, nutrition

## Abstract

As a newly identified manganese transport protein, ZIP14 is highly expressed in the small intestine and liver, which are the two principal organs involved in regulating systemic manganese homeostasis. Loss of ZIP14 function leads to manganese overload in both humans and mice. Excess manganese in the body primarily affects the central nervous system, resulting in irreversible neurological disorders. Therefore, to prevent the onset of brain manganese accumulation becomes critical. In this study, we used *Zip14^−/−^* mice as a model for ZIP14 deficiency and discovered that these mice were born without manganese loading in the brain, but started to hyper-accumulate manganese within 3 weeks after birth. We demonstrated that decreasing manganese intake in *Zip14^−/−^* mice was effective in preventing manganese overload that typically occurs in these animals. Our results provide important insight into future studies that are targeted to reduce the onset of manganese accumulation associated with ZIP14 dysfunction in humans.

## 1. Introduction

ZIP14 (encoded by *SLC39A14* gene) is a newly identified manganese transporter with high expression levels in the small intestine and liver [1,2,3,4,5,6,7], which are two primary organs involved in regulating systemic manganese homeostasis—the intestine controls dietary manganese absorption, whereas the liver clears manganese from the blood and secretes this metal as a bile conjugate for fecal excretion or intestinal reabsorption [8]. Patients carrying *ZIP14* mutations developed neurodegenerative phenotypes with early-onset dystonia due to manganese hyperaccumulation in the brain. These individuals did not accumulate manganese in the liver and had normal liver function [9,10,11]. Consistent with the human phenotype associated with *ZIP14* mutations, *zip14*-deficiency zebrafish hyper-accumulated manganese in the brain, but not in the liver, and presented with reduced locomotor activities [10]; whole body *Zip14* knockout (*Zip14^−/−^*) mice markedly increased manganese in the blood and brain, resulting in impaired locomotor behavior [12,13,14], but had decreased liver manganese [12,14].

Tissue-specific inactivation of *Zip14* in mice further revealed its essential role in regulating manganese metabolism of the liver and intestine. Under normal dietary conditions, liver-specific *Zip14* knockout mice (*Zip14*-L-KO) had significantly decreased manganese in the liver, indicating a critical function for ZIP14 to import manganese to hepatocytes. However, even with reduced manganese uptake into the liver, *Zip14*-L-KO mice had normal manganese levels in the blood and other body tissues, suggesting that hepatic ZIP14 is not the primary control for systemic manganese homeostasis at physiological conditions [13,15]. In contrast to *Zip14*-L-KO mice that did not develop manganese overload in the body, mice with intestinal specific *Zip14* knockout (*Zip14*-In-KO) developed increased manganese in both the liver and brain under normal dietary conditions, highlighting the importance of intestinal ZIP14 in maintaining systemic manganese homeostasis [15,16]. Taken together, these previous studies have confirmed an indispensable role for ZIP14 in regulating manganese homeostasis, because lack of ZIP14 leads to increased manganese absorption through the intestine [15] and impaired manganese clearance through hepatobiliary excretion [10,12,13,14]. Both alterations contribute to the development of manganese overload observed in individuals lacking functional ZIP14.

Manganese overload predominantly affects the central nervous system, resulting in neurological disorders [17,18]. The symptoms of manganese neurotoxicity, once evident, are usually irreversible and continue to progress even after the removal of excess manganese [19]. Therefore, developing proper strategies to prevent the onset of brain manganese overload becomes the critical intervention point. Since *Zip14^−/−^* mice recapitulate the key symptoms of patients carring *ZIP14* mutations, the present study aimed to use *Zip14^−/−^* mice as a model to determine whether brain manganese accumulation caused by loss of ZIP14 could be effectively prevented.

## 2. Results

### 2.1. Zip14^−/−^ Mice Are Born without Manganese Overload

**Figure 1:**
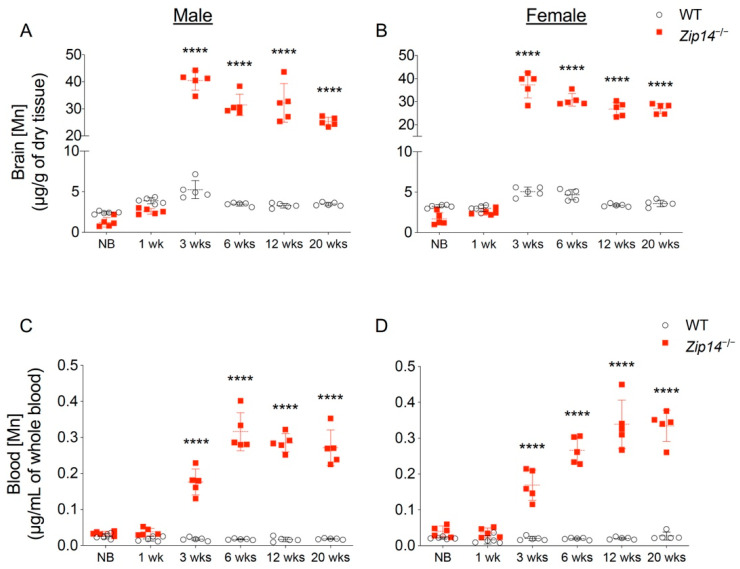
Brain and blood manganese concentrations in mice from different age groups of wild-type (WT) and *Zip14* knockout (*Zip14^−/−^*) mice. (**A**,**B**) Brain and (**C**,**D**) blood manganese (Mn) contents measured by inductively coupled plasma mass spectrometry (ICP-MS) in male and female mice at newborn (NB), 1 week (1 wk), 3 weeks (3 wks), 6 weeks (6 wks), 12 weeks (12 wks) and 20 weeks (20 wks) of age (*n* = 5/group). Data were expressed as mean ± standard deviation (S.D.). Statistical analysis was performed using two-way ANOVA, followed by the Bonferroni post-hoc test to compare the age and sex-matched WT and *Zip14^−/−^* mice. **** *p* < 0.0001.

In order to characterize the time of onset and progression of manganese accumulation, we first measured brain metal contents in different age groups of wild-type and *Zip14^−/−^* mice by ICP-MS analysis. In contrast to previous studies that reported manganese hyperaccumulation in the brain of *Zip14^−/−^* mice at ages older than 4 weeks [12,13,14,20], our results indicated that brain manganese was about 10–15% lower in newborn and 1-week-old *Zip14^−/−^* mice compared to that of the sex and age-matched wild-type littermates (Figure 1A,B). However, at 3 weeks old (the age of weaning), *Zip14^−/−^* mice had approximately 10-fold increased brain manganese compared to the control animals (Figure 1A,B), which is consistent with the results from a previous study using *Zip14^−/−^* mice at 4 weeks old [12]. Since whole-blood manganese can be used as an indicator of body manganese status [21], we next examined the blood manganese contents. Similar to the pattern of brain manganese, blood manganese concentrations in *Zip14^−/−^* mice were not significantly different from that of the wild-type animals before the age of 1 week, but were significantly elevated at 3 weeks old (Figure 1C,D), indicating that *Zip14^−/−^* mice were born without manganese loading. This novel finding led to our hypothesis that interventions introduced early in life may be able to prevent the onset of manganese accumulation caused by ZIP14 deficiency.

### 2.2. Metal Chelation Approach Using CaNa_2_EDTA Cannot Prevent Brain Manganese Loading in Zip14^−/−^ Mice

**Figure 2:**
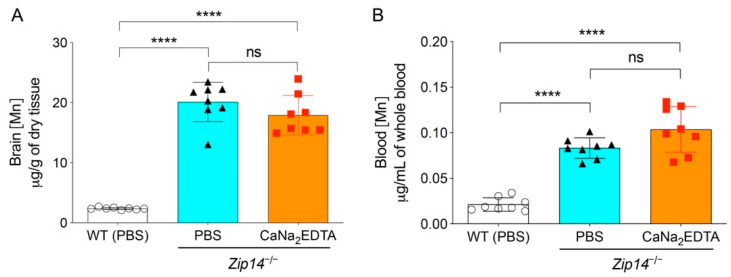
Metal chelation did not prevent the development of brain manganese overload. CaNa_2_EDTA was injected intraperitoneally into 1-week-old *Zip14^−/−^* mice every other day for 2 weeks. Animals were sacrificed and tissues were collected when mice were 3 weeks old. Phosphate buffered saline (PBS) was used as the vehicle control. (**A**) Brain and (**B**) blood manganese concentrations were determined by ICP-MS analysis (*n* = 8, 4 males and 4 females). Data were expressed as mean ± S.D. Statistical analysis was performed using one-way ANOVA, followed by the Bonferroni post-hoc test. **** indicates *p* < 0.0001; “ns” indicates not significant.

The therapy with chelating agent CaNa_2_EDTA is a commonly used clinical approach to alleviate the symptoms of manganese intoxication in human patients [22,23,24]. A previous study showed that in 5-month-old *Zip14^−/−^* mice, intraperitoneal injection of CaNa_2_EDTA (1 mmol/kg body weight) four times a week for a total of four weeks reduced serum manganese levels by approximately 50% [13]. To assess whether the chelation approach could be used in younger animals to prevent the occurrence of brain manganese accumulation, we administered CaNa_2_EDTA intraperitoneally to 1-week-old *Zip14^−/−^* pups every other day for a 2-week period (at a dose of 1 mmol/kg body weight/injection for a total of seven injections) and collected animal tissues at 3 weeks of age. Mice injected with PBS were used as the controls. Since manganese levels in the blood, brain and liver were not statistically different between sexes at 1 week or 3 weeks of age, we used both male and female mice and combined the results for further analysis. Consistent with the results from mice without PBS administration, at 3 weeks of age, the concentrations of brain manganese in PBS-injected *Zip14^−/−^* mice were approximately 10-fold higher than that of PBS-injected wild-type mice (Figure 2A). When comparing with PBS-injected *Zip14^−/−^* mice, CaNa_2_EDTA treatment did not significantly affect the brain and blood manganese (Figure 2A,B), indicating that metal chelation is not effective in preventing brain manganese overload in *Zip14^−/−^* mice.

### 2.3. Hepatic ZIP14 Restoration Increases Liver Manganese, but Does Not Effectively Prevent the Accumulation of Manganese in the Brain

**Figure 3:**
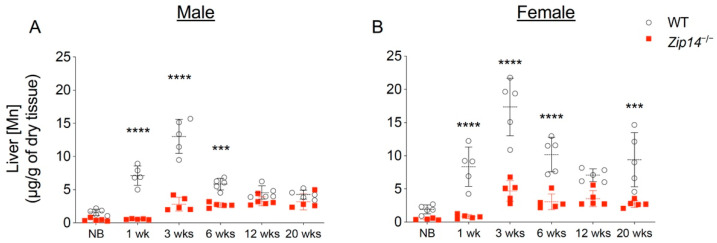
Liver manganese in mice from different age groups of WT and *Zip14^−/−^* mice. Liver manganese concentrations measured by ICP-MS in (**A**) male and (**B**) female mice at newborn (NB), one week (1 wk), three weeks (3 wks), six weeks (6 wks), twelve weeks (12 wks) and twenty weeks (20 wks) of age (*n* = 4–5/group). Data were expressed as mean ± S.D. and statistical analysis was performed using two-way ANOVA, followed by the Bonferroni post-hoc test to compare results of WT and *Zip14^−/−^* mice from the same age group. *** *p* < 0.001; **** *p* < 0.0001.

The liver plays an important role in regulating hepatobiliary manganese excretion. Although *Zip14*-L-KO mice did not develop manganese overload under normal conditions, when challenged with a high-manganese diet, *Zip14*-L-KO mice accumulated about two times more manganese in the brain and blood compared to the control animals [13], suggesting that hepatic ZIP14 is required for efficient clearance of excessive manganese under high-manganese conditions [13]. Compared to the wild-type animals, *Zip14^−/−^* mice had a 60–80% reduction in liver manganese at 1 week and 3 weeks of age, indicating that ZIP14 is the major manganese importer in the liver during early growth (Figure 3A,B). At 1 week old, *Zip14^−/−^* mice did not accumulate manganese in the brain (Figure 1A,B). At 3 weeks old, however, when *Zip14^−/−^* mice presented with about 10 times manganese in the blood, they accumulated around 10 times as much manganese in the brain as the wild-type controls, suggesting an inadequate clearance of excess manganese from the circulation. We reason that at around 1 week old—before manganese accumulation occurs in the brain—if the ZIP14 protein is reintroduced into the liver, it may reduce circulating manganese by facilitating hepatobiliary excretion and may avoid systemic manganese overload occurred at a later life stage in *Zip14^−/−^* mice.

**Figure 4:**
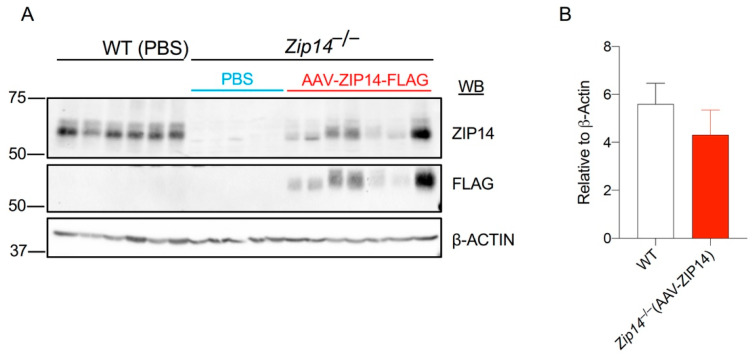
Adeno-associated virus (AAV)-mediated hepatic ZIP14 expression in *Zip14^−/−^* mice. *Zip14^−/−^* mice received a single dose of ZIP14-expressing AAV (AAV-ZIP14-FLAG) via intraperitoneal injection at 1 week old. At 3 weeks of age, mice were sacrificed and tissue samples were collected. PBS was used as the vehicle control. (**A**) Liver samples were analyzed by Western blotting (WB). Blots were probed for ZIP14 using both anti-mouse ZIP14 and anti-FLAG antibodies. β-Actin was used as a loading control. (**B**) The relative expression levels of ZIP14 in PBS-injected WT mice and AAV-ZIP14-injected *Zip14^−/−^* mice were determined by normalizing to β-ACTIN (*n* = 5–7 female mice/group).

In order to develop an animal model for hepatic ZIP14 restoration, we inserted the sequence encoding mouse ZIP14, followed by a FLAG epitope, into a hepatocyte specific AAV8 vector. The addition of a FLAG sequence enables the differentiation between endogenous ZIP14 and reintroduced ZIP14 by the anti-FLAG antibody. We aimed to use this AAV8-mediated gene-delivery approach to test the effect of hepatic ZIP14 reintroduction because this approach facilitates the protein expression at a close-to-physiologic level and does not stimulate inflammatory responses in tested animals, as evidenced by previous studies [25,26]. At 1 week old, *Zip14^−/−^* mice received a single dose of AAV8-ZIP14 via intraperitoneal injection. At 3 weeks of age, mice were sacrificed and tissue samples were collected. Immunoblotting by the anti-FLAG antibody confirmed the AAV8-mediated delivery of ZIP14 into the liver; while the same assay by the anti-ZIP14 antibody allowed the comparison of ZIP14 expression between wild-type and AAV8-injected *Zip14^−/−^* mice (Figure 4A, Supplementary Figure S1). Quantification of the ZIP14 immunoreactive bands revealed ZIP14 restoration to about 80% of the wild-type level (Figure 4B).

**Figure 5:**
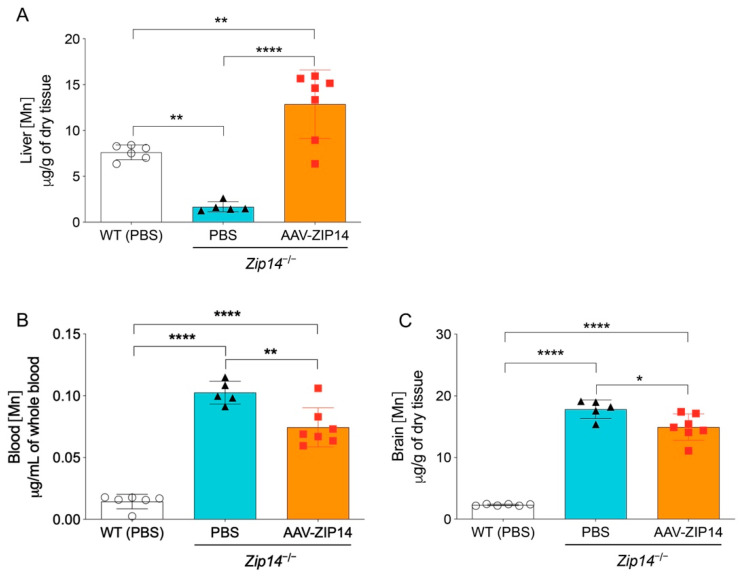
Restoration of hepatic ZIP14 expression increased liver manganese levels, but did not prevent brain manganese accumulation. (**A**) Liver, (**B**) blood and (**C**) brain manganese concentrations in PBS-injected WT and *Zip14^−/−^* mice and AAV-ZIP14 injected *Zip14^−/−^* mice were determined by ICP-MS (*n* = 5–7 female mice/group). Data were expressed as mean ± S.D. and statistical analysis was performed using one-way ANOVA, followed by the Bonferroni post-hoc test. * *p* < 0.05; ** *p* < 0.01 and **** *p* < 0.0001.

ICP-MS assessment indicated that the restoration of hepatic ZIP14 significantly increased liver manganese in *Zip14^−/−^* mice, demonstrating the efficacy of AAV-mediated ZIP14 expression (Figure 5A). In the meantime, when comparing to the vehicle-injected *Zip14^−/−^* mice, the AAV-injected *Zip14^−/−^* mice had approximately 20% and 10% reduced manganese in the blood and brain, respectively. However, compared to vehicle-injected wild-type mice, *Zip14^−/−^* mice with hepatic ZIP14 reintroduction still accumulated between six and seven times manganese in the brain and blood (Figure 5B,C). These results indicated that although ZIP14 restoration in the liver could reduce the extent of manganese loading by facilitating the import of manganese into the liver, this approach was not effective in preventing the occurrence of brain manganese hyperaccumulation in *Zip14^−/−^* mice.

### 2.4. Maternal Milk Manganese Level Can Be Altered by Dietary Intervention

**Figure 6:**
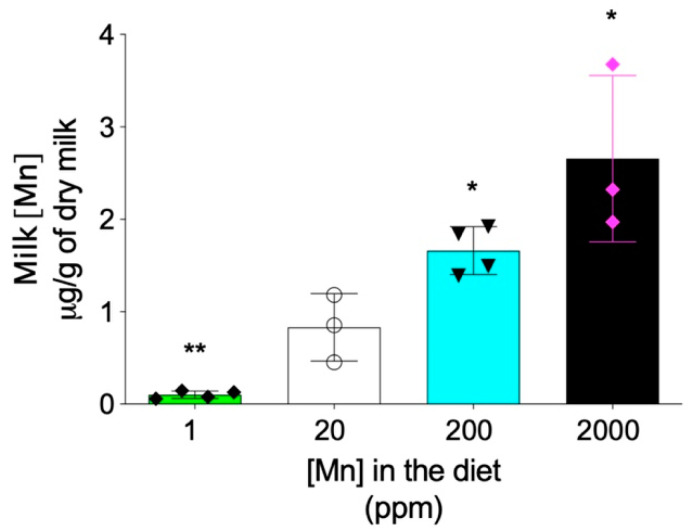
Maternal breast milk manganese content can be altered by dietary intervention. *Zip14^+^*^/−^ mating mice at 8–9 weeks old were fed diets containing 1 ppm, 20 ppm, 200 ppm, or 2000 ppm of manganese during breeding and lactation periods (for a total of ~6–7 weeks). Milk manganese contents were analyzed by ICP-MS (*n* = 3–4/group). Data were expressed as mean ± S.D. and statistical analysis was performed using one-way ANOVA, followed by the Bonferroni post-hoc test. * *p* < 0.05; ** *p* < 0.01, compared with the control group (20 ppm).

In addition to the hepatobiliary excretion route, the intestinal absorption also plays important role in regulating the systemic manganese homeostasis [8,27]. Previous studies have demonstrated that, differently from *Zip14*-L-KO mice that did not develop systemic manganese overload, *Zip14*-In-KO mice had increased manganese in both the liver and brain under normal dietary conditions [15,16], suggesting that lack-of-ZIP14 increases intestinal manganese absorption [15]. Therefore, we next sought to determine whether limiting manganese intake can prevent the development of manganese overload in *Zip14^−/−^* mice. Since maternal milk is the primary nutrient source for neonatal mice before weaning at 3 weeks of age, we first examined whether manganese content in mouse breast milk can be altered through dietary intervention. We fed mating mice (*Zip14^+/−^* mice, 8–9 weeks old) with modified AIN-93G diets containing four levels of manganese: 1 ppm, 20 ppm, 200 ppm and 2000 ppm. The 20 ppm manganese diet was used as the control for two reasons: first, the estimated dietary manganese requirement for mice is 10 ppm and, second, consumption of diets containing 5 ppm or 45 ppm manganese during gestation and lactation did not result in differences in tissue manganese levels or manganese-containing enzyme activities in both maternal and weanling mice [28]. The 2000 ppm was used as the highest dietary manganese level, because a previous study demonstrated that diet containing 2400 ppm of manganese did not result in toxicity in mice [29]. ICP-MS analysis revealed that manganese content in breast milk varied in gradient with different diets, with decreased dietary intake resulting in a lower level of manganese in the breast milk (Figure 6).

### 2.5. Early Intervention to Restrict Manganese Intake Prevents the Onset of Brain Manganese Loading in Zip14^−/−^ Mice

**Figure 7:**
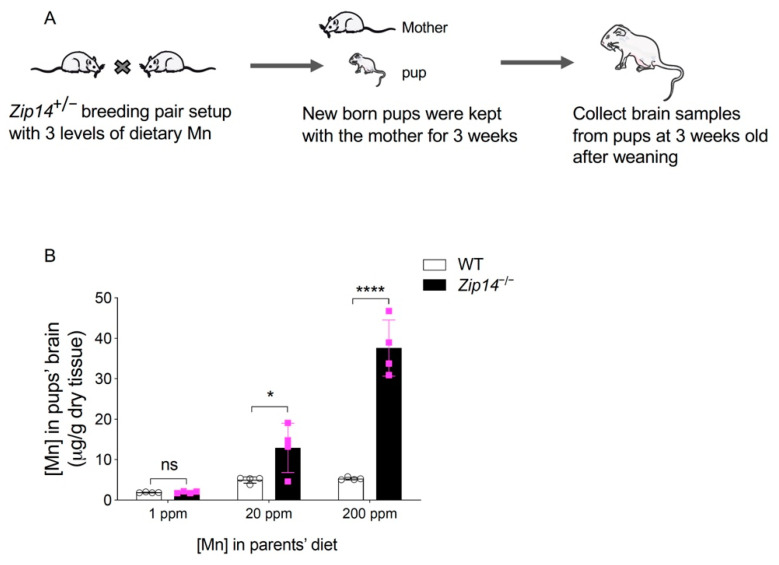
Restricting manganese intake prevents the onset of brain manganese loading in *Zip14^−/−^* mice. (**A**) Schematic illustrating the experimental procedures. Heterozygous *Zip14*^+/−^ mating mice were fed with diets containing low to normal levels of Mn (1 ppm, 20 ppm and 200 ppm). Maternal mice were kept on the same diet during the mating and lactation period. Pups were sacrificed and brain tissues were collected at 3 weeks old after weaning. (**B**) Brain manganese was determined by ICP-MS analysis (*n* = 4/group, 2 males and 2 females). Data were expressed as mean ± S.D. and statistical analysis was performed using two-way ANOVA, followed by the Bonferroni post-hoc test to compare results of WT and *Zip14^−/−^* mice. * *p* < 0.05; **** *p* < 0.0001; “ns” indicates not significant.

Having established that the manganese content in breast milk can be altered through dietary intervention, we then examined whether restricting manganese intake can prevent the development of brain manganese overload in *Zip14^−/−^* mice. Heterozygous *Zip14*^+/*−*^ mating mice were fed with a diet containing 1 ppm, 20 ppm, or 200 ppm of manganese. Maternal mice were kept on the same diet during the whole mating and lactation period. Pups were sacrificed and brain tissues were collected at 3 weeks old after weaning (Figure 7A). Brain manganese contents were measured by ICP-MS. In the pups born to the 200 ppm manganese-fed dams, *Zip14^−/−^* mice accumulated manganese to the level that was approximately 10 times control (Figure 7B), which is consistent with the results indicated in Figure 1, showing that *Zip14^−/−^* mice fed the traditional rodent NIH 31 diet (155 ppm of manganese) developed 10-fold manganese loading in the brain at the same age. Importantly, *Zip14^−/−^* pups born to dams fed diet containing 1 ppm manganese had similar levels of brain manganese compared to that of the wild-type littermates (Figure 7B). These results demonstrated that limiting manganese intake at an early life stage can effectively prevent manganese hyperaccumulation caused by ZIP14 deficiency. 

## 3. Discussion

As an essential trace element, manganese is required for various physiological processes, including antioxidant defense, protein glycosylation, urea formation and gluconeogenesis [8,27,30,31]. Excess manganese in the body, however, is a potent neurotoxicant and results in the clinical condition known as manganism [32,33]. Neuronal damage induced by excess manganese is typically irreversible. Therefore, to prevent the onset of brain manganese accumulation becomes a challenging topic in manganese overload-related neurological disease. 

Mutations in *ZIP14* have been recognized as one major cause of recessively inherited manganese overload disorder [9,10,11,34,35]. During early childhood, individuals with homozygous loss-of-function alleles in the *ZIP14* gene are manifested with neurodegenerative symptoms and progressive dystonia due to manganese hyperaccumulation in the brain. However, it is important to note that in the reported human *ZIP14* mutation cases, affected individuals appeared normal with average neurodevelopment in the neonatal period (in the first 4 weeks after birth, or even until several months old), suggesting that these individuals did not have excess brain manganese at a very early life stage. 

In the present study, we used *Zip14^−/−^* mice as an animal model for ZIP14 deficiency to determine the time of onset and progression of manganese loading and to investigate whether the brain manganese accumulation could be prevented in these animals. Age-dependent tissue metal analyses revealed that *Zip14^−/−^* mice were born without manganese loading in the brain. However, these mice developed manganese hyperaccumulation by 3 weeks old. These results provided fundamental information on the time of onset and development of manganese overload in *Zip14^−/−^* mice. Based on these novel findings, we further investigated the efficacy of three strategies, namely, metal chelation, hepatic ZIP14 restoration and restriction of manganese intake in preventing the development of brain manganese accumulation. We presented evidence to demonstrate that among these three approaches, decreasing manganese intake in newborn *Zip14^−/−^* mice effectively prevented the manganese overload that typically occurred at a later life stage in these animals. Future behavior tests would be needed to determine whether early dietary manganese restriction can effectively prevent the development of neurological phenotypes associated with loss of ZIP14. 

In summary, our present study focused on preventing the onset of brain manganese loading. Our results could be helpful for future studies aiming to implement intervention studies to reduce disease onset and may provide important insights into the development of therapeutic strategies for manganese overload disorders caused by the loss of ZIP14 function.

## 4. Materials and Methods

### 4.1. Animals, Genotyping and Tissue Collection

Procedures for animal experiments were approved by the Institutional Animal Care and Use Committee of the University of Arizona. Animal cages containing less than 5 mice were kept at 21–22 °C with 12 h of light/dark cycles. Mice were provided with tap water ad libitum and fed a NIH-31 irradiated traditional rodent diet (Teklad 7913; Envigo, Indianapolis, IN, USA). *Zip14* knockout (*Zip14^−/−^*) mice were purchased from Mutant Mouse Resource and Research Centers, USA. *Zip14^−/−^* mice and the wild-type control littermates were generated by heterozygous breeding. Mouse Direct PCR kit (Bimake, Houston, TX, USA) and the following primers were used to determine mouse genotypes: DNA506-100, 5′-TCA TGG ACC GCT ATG GAA AG-3′; DNA506-101, 5′-GTG TCC AGC GGT ATC AAC AGA GAG-3′; Neo3a, 5′-GCA GCG CAT CGC CTT CTA TC-3′; DNA506-6, 5′-TGC CTG GCA CAT AGA ATG C-3′. Mice were sacrificed after anaesthetizing with ketamine/xylazine at a specified age. Blood samples were collected via cardiac puncture into EDTA-containing tubes. Mouse milk samples were collected from the stomach of new born pups. Organ samples were collected and immediately frozen in liquid nitrogen and stored at −80 °C until further analysis.

### 4.2. AAV Production and Injection

The adeno-associated virus (AAV8) vector driven by a liver-specific promoter has been described previously [25,26]. To generate ZIP14-expressing AAV vector (AAV-ZIP14), the coding sequence of mouse ZIP14 (mZIP14) was amplified by PCR from pCMV-Entry-mZIP14 plasmid (Origene, Rockville, MD, USA), using the following primer set: forward, 5′-AAT ATG GTA CCA TGA AGC GGC TGC ACC C-3′; reverse, 5′-ATA TTG CTA GCG CGG CCG CCT ATT TAT CGT C-3′. The PCR product was purified from a 2% agarose gel using the Wizard SV Gel and PCR clean-up system (Promega, Madison, WI, USA) and inserted into KpnI-NheI sites of the AAV vector. AAV particles were produced at the Molecular Virology Core Facility of the Oregon Health and Science University. The AAV stocks were handled according to Biohazard Safety Level 2 guidelines published by the National Institutes of Health. For AAV administration into mice, 1 × 10^13^ viral genomes (vg) of AAV-ZIP14 were prepared in 250 µL of sterile phosphate buffered saline (PBS) and injected intraperitoneally at the age of 8 days. Control mice were injected with 250 µL of sterile PBS solution. Mice were sacrificed at 22 days of age.

### 4.3. Dietary Intervention and Chelator Injection

For dietary intervention, mice were fed traditional rodent diet until the breeding pair setup at around 8–9 weeks old. Then the mating mice were fed one of four AIN-93G purified animal diets modified to contain 1 ppm, 20 ppm, 200 ppm or 2000 ppm manganese (Envigo, Indianapolis, IN, USA), representing low to high levels of manganese. For chelation intervention, the metal chelator, calcium disodium EDTA (CaNa_2_EDTA) (MilliporeSigma, St. Louis, MO, USA) was prepared in PBS and sterilized through a 0.22 µm filter (VWR, Radnor, PA, USA) to make a 0.2 M stock solution. Intraperitoneal chelator injections were carried out at the age of 8 days with a dose of 1 mmol/kg/day as reported previously [13,36]. An equal volume of PBS was injected into vehicle control groups. The injection was administered every other day for 15 consecutive days (a total of 8 injections). Mice were sacrificed at the age of 22 days (considered as 3 weeks old).

### 4.4. Tissue Metal Analysis by Inductively Coupled Plasma Mass Spectrometry (ICP-MS)

Whole blood samples (50 μL) were added to 1.95 mL 3% HNO_3_ and incubated at 85 °C for 4 h. After digestion, samples were centrifuged at 2000× *g* for 10 min and the supernatant was collected for further analysis. Frozen animal tissues were thawed and dried in an oven at 80 °C for at least 2 days until they reached a constant weight. Dried tissues were digested with 1 mL of concentrated HNO_3_ at room temperature (RT) overnight and then at 60 °C for 24 h. Digests were dried at 80 °C in an oven. Each dried sample was subsequently re-dissolved in 9 mL of 3% HNO_3_ as the final digests. The samples from age-dependent and dietary intervention experiments were prepared at the Arizona Laboratory for Emerging Contaminants. The samples from AAV injection and metal chelation experiments were prepared separately in our laboratory following the same procedure. Metal levels of the final digests were analyzed by ICP-MS at the Arizona Laboratory for Emerging Contaminants using the Agilent 7700× ICP-MS instrument (Agilent Technologies, Santa Clara, CA, USA). The instrument parameters for manganese were as follows: radio frequency power—1550 W; carrier gas flow rate and mode—1.0 L/min, He mode (collision gas) with gas flow at 0.5 mL/min; plasma gas flow rate—15 L/min; auxiliary gas flow rate—0.90 L/min. The analytical QA/QC protocol was adapted from US EPA Method 200.8 for analysis by ICP-MS. Calibration standards were prepared from multi-element stock solution (SPEX CertiPrep, Metuchen, NJ, USA). Calibration curves include at least 7 points with correlation coefficients >0.995. The QC protocol includes a continuing calibration blank, a continuing calibration verification solution and at least one quality control sample to be analyzed just after calibration and again after 12 samples and at the completion of all sample analysis. Acceptable QC responses must be between 90% and 100% of the certified value.

### 4.5. Immunoblotting Analysis

Tissues samples were lysed in NETT buffer (150 mM NaCL, 5 mM EDTA, 10 mM Tris, 1% TritonX-100, 1× Protease inhibitor cocktail (Bimake, Houston, TX, USA)). Protein concentrations of the lysates were determined by the *RC DC* assay kit (Bio-Rad Life Science, Hercules, CA, USA). Tissue lysates with 55 µg of protein were mixed with 1× Laemmli buffer and incubated at 37 °C for 30 min. Proteins were electrophoretically separated on sodium dodecyl sulfate (SDS)/10% polyacrylamide gels and transferred to nitrocellulose membranes (GVS, Sanford, ME, USA). After membranes were incubated for 1 h with blocking buffer (5% non-fat dry milk in TBST (10 mM Tris/HCl, 150 mM NaCl, 0.1% 1 mL Tween-20, pH 7.50)) at RT, followed by rabbit anti-mouse ZIP14 antibody (1:1000) overnight at 4 °C, membranes were washed 4 times with TBST (5 min/each) and incubated for 1 h at RT with donkey anti-rabbit horseradish peroxidase (HRP)-conjugated secondary antibody (1:3000) (GE healthcare, Chicago, IL, USA). Blots were washed twice (5 min each) with TBST, followed by two washes in TBS prior to imaging. In addition, since AAV-ZIP14 contains a FLAG epitope, a mouse monoclonal HRP-conjugated anti-FLAG antibody (1:6000) (Sigma-Aldrich, St. Louis, MO, USA) was used to detect FLAG-tagged ZIP14 to differentiate AAV-ZIP14 and endogenous ZIP14 in mouse tissues. Blots were developed using enhanced chemiluminescence (SuperSignal West Pico, Thermo Fisher Scientific, Waltham, MA, USA) and the ChemiDoc MP Imaging System (Bio-Rad Life Science). To confirm equivalent loading, blots were stripped for 15 min in Restore PLUS Western Blot Stripping Buffer (Thermo Fisher Scientific), washed 4 times in TBS (5 min each), blocked for 1 h in blocking buffer and re-probed with HRP-conjugated anti-β-ACTIN (1:10,000) antibody (Proteintech, Rosemont, IL, USA).

### 4.6. Statistical Analysis

Data were analyzed by one-way or two-way ANOVA for multiple group comparisons with Prism 8.0 (GraphPad Software, San Diego, CA, USA). Bonferroni post-hoc tests were performed for multiple comparisons. Differences with *p* value <0.05 were considered to be statistically significant. 

## Data Availability

The data presented in this study are available on request from the corresponding author.

## Supplementary Materials

The following is available online at https://www.mdpi.com/article/10.3390/ijms22136773/s1, Figure S1: Uncropped Western blot images shown in Figure 4A.
