# Supplementary material for: Restriction of Manganese Intake Prevents the Onset of Brain Manganese Overload in Zip14−/− Mice
Source: Int J Mol Sci. 2021 Jun 24;22(13):6773. doi: 10.3390/ijms22136773 (PMC8268934; doi:10.3390/ijms22136773)
Supplement: Supplementary file 1 [file ijms-22-06773-s001.zip › ijms-1249074-supplementary.pdf]

Supplementary Material

Restriction of Manganese Intake Prevents the Onset of Brain Manganese Overload in *Zip14*$^{-/-}$ Mice

Yuze Wu, Guojun Wei and Ningning Zhao *

Department of Nutritional Sciences, The University of Arizona, Tucson, AZ 85721, USA; yuzewu@email.arzona.edu (Y.W.); gwei@email.arizona.edu (G.W.)

* Correspondence: zhaonn@email.arizona.edu; Tel.: +1-520-621-9744

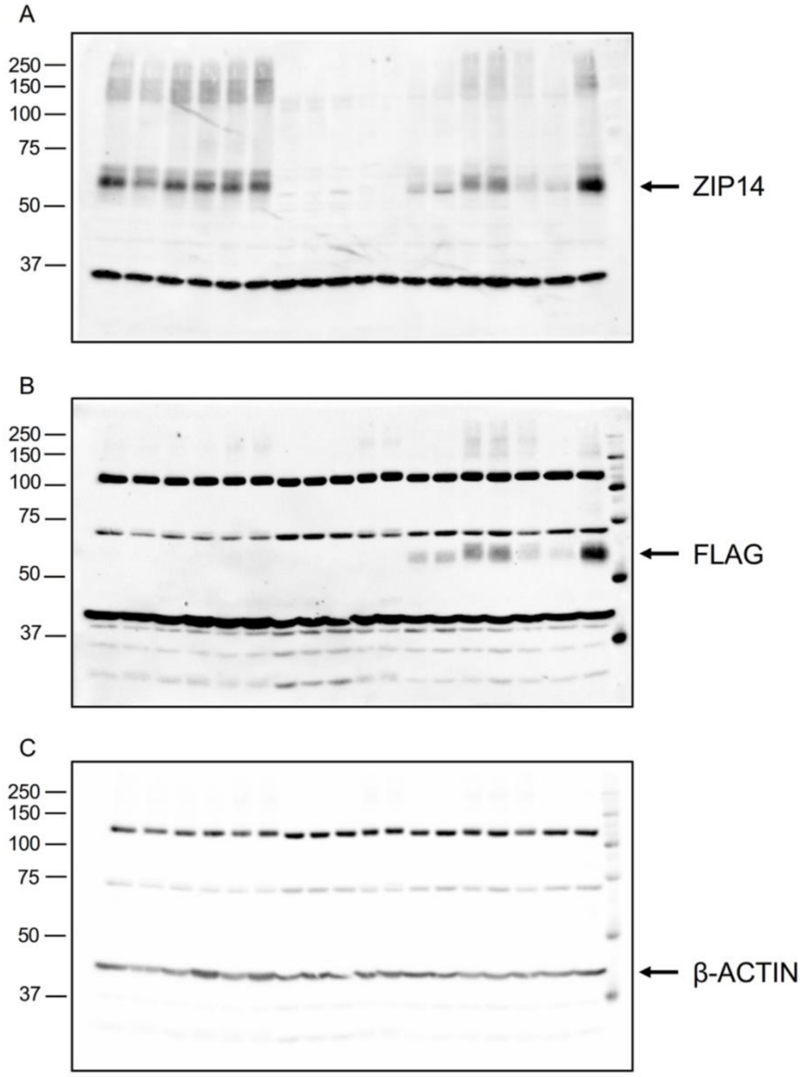

Figure S1. Uncropped Western blot images shown in Figure 4A. Uncropped images for (**A**) ZIP14 blot, (**B**) FLAG blot, and (**C**) β-ACTIN blot.
